# Transcriptomic Regulation of Muscle Mitochondria and Calcium Signaling by Insulin/IGF-1 Receptors Depends on FoxO Transcription Factors

**DOI:** 10.3389/fphys.2021.779121

**Published:** 2022-02-04

**Authors:** Gourav Bhardwaj, Christie M. Penniman, Katherine Klaus, Eric T. Weatherford, Hui Pan, Jonathan M. Dreyfuss, K. Sreekumaran Nair, C. Ronald Kahn, Brian T. O’Neill

**Affiliations:** ^1^Fraternal Order of Eagles Diabetes Research Center and Division of Endocrinology and Metabolism, Roy J. and Lucille A. Carver College of Medicine, University of Iowa, Iowa City, IA, United States; ^2^Division of Endocrinology and Metabolism, Mayo Clinic College of Medicine and Science, Rochester, MN, United States; ^3^Bioinformatics and Biostatistics Core, Joslin Diabetes Center, Harvard Medical School, Boston, MA, United States; ^4^Section on Integrative Physiology and Metabolism, Joslin Diabetes Center, Harvard Medical School, Boston, MA, United States; ^5^Veterans Affairs Health Care System, Iowa City, IA, United States

**Keywords:** insulin/IGF-1 receptors, FoxO transcription factors, muscle transcription, diabetes, RNA sequencing, mitochondrial dysfunction, calcium signaling

## Abstract

Insulin and IGF-1, acting through the insulin receptor (IR) and IGF-1 receptor (IGF1R), maintain muscle mass and mitochondrial function, at least part of which occurs via their action to regulate gene expression. Here, we show that while muscle-specific deletion of IR or IGF1R individually results in only modest changes in the muscle transcriptome, combined deletion of IR/IGF1R (MIGIRKO) altered > 3000 genes, including genes involved in mitochondrial dysfunction, fibrosis, cardiac hypertrophy, and pathways related to estrogen receptor, protein kinase A (PKA), and calcium signaling. Functionally, this was associated with decreased mitochondrial respiration and increased ROS production in MIGIRKO muscle. To determine the role of FoxOs in these changes, we performed RNA-Seq on mice with muscle-specific deletion of FoxO1/3/4 (M-FoxO TKO) or combined deletion of IR, IGF1R, and FoxO1/3/4 in a muscle quintuple knockout (M-QKO). This revealed that among IR/IGF1R regulated genes, >97% were FoxO-dependent, and their expression was normalized in M-FoxO TKO and M-QKO muscle. FoxO-dependent genes were related to oxidative phosphorylation, inflammatory signaling, and TCA cycle. Metabolomic analysis showed accumulation of TCA cycle metabolites in MIGIRKO, which was reversed in M-QKO muscle. Likewise, calcium signaling genes involved in PKA signaling and sarcoplasmic reticulum calcium homeostasis were markedly altered in MIGIRKO muscle but normalized in M-QKO. Thus, combined loss of insulin and IGF-1 action in muscle transcriptionally alters mitochondrial function and multiple regulatory and signaling pathways, and these changes are mediated by FoxO transcription factors.

## Introduction

Both type 1 and type 2 diabetes are associated with decreased muscle strength and quality, which can compromise overall fitness and leads to increased mortality (Park et al., 2006; Barzilay et al., 2009; Orlando et al., 2017). Various mechanisms contribute to muscle weakness in diabetes including impaired protein turnover (James et al., 2017), decreased mitochondrial function (Kelley et al., 2002; Mogensen et al., 2007; Ruegsegger et al., 2018; Bhardwaj et al., 2021), and increased oxidative stress (Henriksen et al., 2011), many of which are regulated by transcriptional mechanisms. Insulin is a pleiotropic anabolic hormone and is known to regulate a variety of transcriptional pathways in muscle. Previous studies in mice and humans show that insulin treatment, under euglycemic clamp conditions, modulates up to 1000 transcripts in muscle, including genes related to mitochondrial function, autophagy, glucose and lipid utilization, cytoskeletal organization, protein turnover, chromatin remodeling, and mRNA splicing (Rome et al., 2003; Batista et al., 2019), but the transcription factors that mediate these effects are not well defined.

Insulin and IGF-1 act through insulin receptors (IR) and highly homologous IGF-1 receptors (IGF1R) and regulate metabolic response via IRS-1/phosphatidylinositol 3-kinase (PI3K)/AKT pathways, and growth and differentiation through SHC/RAS/mitogen-activated protein kinase (MAPK) pathways (Taniguchi et al., 2006; James et al., 2017; Haeusler et al., 2018). The most well-described insulin-regulated transcription factors are Forkhead box O proteins (FoxO). FoxOs are highly conserved transcription factors that are phosphorylated and inactivated by Akt (Guo et al., 1999; Nakae et al., 1999), and FoxOs 1, 3, and 4 are highly expressed in muscle (O’Neill et al., 2016). Muscle-specific transgenic overexpression of FoxO1 has been shown to decrease cytoskeletal genes and induce genes related to ubiquitin-proteasome and lysosomal degradation, but effects on other pathways is not well studied (Kamei et al., 2004; Stitt et al., 2004). In cardiomyocytes, FoxO3 regulates cytosolic and mitochondrial calcium homeostasis (Lu et al., 2013; Chaanine et al., 2016). FoxOs also respond to various stress conditions, and in this context have been shown to regulate genes of autophagy, oxidative stress, cell cycle, and mitochondrial metabolism (Eijkelenboom and Burgering, 2013).

FoxOs are critical mediators of diabetes-related muscle atrophy downstream of IR/IGF1R, and control muscle mass via regulation of ubiquitin-proteasome and autophagy-lysosome pathways (O’Neill et al., 2016, 2019). Studies in mice and humans indicate that insulin deficiency in diabetes decreases mitochondrial respiration, ATP production and downregulates the mitochondrial genes and proteins, which is prevented with the treatment of insulin (Patti et al., 2003; Stump et al., 2003; Yechoor et al., 2004; Mogensen et al., 2007; Zabielski et al., 2016). Recently, we showed that the IR/IGF1R-FoxO signaling axis controls complex-I dependent mitochondrial bioenergetics (Bhardwaj et al., 2021), indicating that inhibition of FoxOs by insulin/IGF-1 action helps maintain muscle mass and mitochondrial metabolism in muscle. The goal of this study is to determine the role of IR/IGF1R mediated repression of FoxOs in regulation of the muscle transcriptome.

In the present study we show that while loss of IR or IGF1R alone only mildly alter gene expression, combined loss of both IR/IGF1R in MIGIRKO mice alters the expression of >3000 genes related to multiple pathways important for muscle function, including fibrosis, mitochondrial function, hypertrophy, protein kinase A signaling and calcium homeostasis. Most of the genes related to mitochondrial function were downregulated in MIGIRKO muscle, and this results in a decrease in mitochondrial respiratory capacity and an accumulation of TCA cycle intermediates, whereas oxidative stressors are increased. We find that the expression of nearly all of the genes altered in MIGIRKO muscle are restored to normal when FoxO transcription factors are deleted in M-QKO mice. When comparing transcriptional changes induced by IR/IGF1R deletion, we find that FoxO-dependent genes comprise > 97% of all changes and include genes related to oxidative phosphorylation, inflammatory signaling, and TCA cycle. Uncovering these insulin- and FoxO- regulated pathways provides insights into mechanism by which diabetes alters muscle metabolic function and new targets for improving muscle strength and preventing disability in patients with diabetes.

## Materials and Methods

### Animal Care and Use

All animals used in this study were approved by the Institutional Animal care and Use Committee (IACUC) at both the University of Iowa and Joslin Diabetes Center. This study included various muscle-specific knock out mouse lines which use Cre expressed by a muscle-specific promoter (Acta1, also known as Human Skeletal Actin [HSA]) crossed with various genes targeted with Lox P sites (lox/lox). For MIGIRKO, M-IR^–/–^ and M-IGF1R^–/–^, the initial cross between an Acta1-Cre (stock 006149; Jackson Laboratory) female on a C57Blk6J background and an IR^*lox*/*lox*^, IGF1R^*lox*/*lox*^ male (mixed C57Blk6, C57Blk6J, and 129 strains) was done in 2011. The F1 and F2 generations were intercrossed and breeders were selected to obtain the three colonies M-IR^–/–^ (Acta1-cre, IR^*lox*/*lox*^), M-IGF1R^–/–^ (Acta1-cre, IGF1R^*lox*/*lox*^), and MIGIRKO (Acta1-cre, IR^*lox*/*lox*^, IGF1R^*lox*/*lox*^). These mice were extensively characterized for muscle mass and glucose homeostasis (O’Neill et al., 2015, 2016; Bhardwaj et al., 2021). In 2013, muscle quintuple knockout and FoxO TKO mice were created by crossing Acta1-Cre, IR^*lox*/+^, IGF1R^*lox*/*lox*^ from the MIGIRKO colony to FoxO1^*lox*/*lox*^, FoxO3^*lox*/*lox*^, FoxO4^*lox*/*lox*^ founder (Mixed C57Blk6, C57Blk6J, and 129 strains) courtesy of Dr. Domenico Accili. M-FoxO TKO and M-QKO were derived by crossing back to FoxO1^*lox*/*lox*^, FoxO3^*lox*/*lox*^, FoxO4^*lox*/*lox*^, then selecting for breeders from this F2 generation, which were again well studied for proteostasis, muscle atrophy (O’Neill et al., 2016), and for mitochondrial function (Bhardwaj et al., 2021). Thus, all animals were on a mixed background containing C57Blk6, C57Blk6J, and 129 strains. Free access to food and water was available to all animals and all animals were euthanized between 9 and 11 AM to minimize nutritional variability.

Given the mixed background of the initial founders and multiple crosses needed to generate these mice, we acknowledge that genomic heterogeneity is a limitation of the study. Importantly, each knockout is compared with their respective littermate ^*lox*/*lox*^ (“floxed”) controls. Thus for the current study, to minimize genetic heterogeneity, M-IR^–/–^ mouse line is compared with IR^*lox*/*lox*^ controls, M-IGF1R^–/–^ is compared with IGF1R^*lox*/*lox*^, MIGIRKO is compared with IR/IGF1R^*lox*/*lox*^, M-FoxO TKO is compared with FoxO T^*lox*/*lox*^, and M-QKO is compared with Q^*lox*/*lox*^ controls. PCA analysis of all strains indicate that the “floxed” controls cluster together, but we cannot exclude that genetic variability may contribute to some of the transcriptomic changes.

All animals were allowed free access of food and water and euthanize at around 9 AM under *ad libitum* fed conditions.

### Transcriptomic Analysis

Transcriptomic profiling was performed on RNA extracted from the mixture of quadriceps and gastrocnemius muscle as described previously (O’Neill et al., 2019; Bhardwaj et al., 2021) and the Biopolymers Facility at Harvard Medical School. All RNA was extracted at the same time, and all sequencing was done together. Briefly, RNA was extracted using Trizol reagent (Invitrogen), submitted to RNA sequencing, and each sample was bioinformatically aligned to the mouse genome using STAR (Dobin et al., 2013), counted, and normalized (Robinson and Oshlack, 2010). To compare each knock-out line with their respective floxed controls, moderated *t*-tests were applied from the limma package (Ritchie et al., 2015) and false discovery rates (FDR) were calculated using the Benjamini-Hochberg method. We defined FoxO dependent genes as those where log2 of the fold change (logFC) of MIGIRKO vs. IR/IGF1R^lox/lox^ was statistically greater than twice the logFC of M-QKO vs. M-FoxO TKO (McCarthy and Smyth, 2009). We defined FoxO independent genes as those where changes in MIGIRKO vs. IR/IGF1R^lox/lox^ and M-QKO vs. M-FoxO TKO were in the same direction and the minimum of the absolute values of the t-statistics was higher than expected by chance. If the t-statistics had opposite signs, they were set to 0. We tested if these minima were larger than simulated minima to obtain *p*-Values, which were used to calculate FDRs. All statistics were performed on R software. Principal component analysis (PCA) plots were generated using plotPCA function from DEseq2 (Love et al., 2014) and ggplot2 package in the R (4.0.3) environment. Volcano plots were prepared in GraphPad Prism software. RNA seq data is publicly available at Gene Expression Omnibus database (GSE178356 and GSE136948).

### Pathway Analysis From Transcriptomic Data

Pathway enrichment analysis was performed using Ingenuity Pathway Analysis (Ingenuity Systems, Redwood City, United States) software. We have removed RIKEN genes from all the analysis for this manuscript because many of these genes were not mapped in the pathway enrichment analysis. Only genes with FDR < 0.05 and fold change ± 1.5 between groups were taken into consideration. For each pathway analysis, Insr (IR), Igf1r, Foxo1, Foxo3, and/or Foxo4 gene expression was/were excluded if they were genetically knocked out in the mouse model used for RNA-Seq. Fisher exact test was used to determine the significantly affected pathways.

### Metabolomics

Tricarboxylic acid (TCA) cycle metabolites were measured in the mixture of quadriceps and gastrocnemius at the Mayo Clinic Metabolomics Resource Core facility using time of-flight mass spectrometer.

### Mitochondrial Respiration

Mitochondrial respiration and the coupling efficiency were measured in the mitochondrial isolates of quadriceps using high resolution respirometer (Oroboros Oxygraph) as previously described (Zabielski et al., 2016). Briefly, mitochondria were isolated from quadriceps muscle with a Dounce homogenizer and differential centrifugation. Respiratory capacity was measured in mitochondrial isolates, and with the sequential addition of 10 mmol/L glutamate/2 mmol/L malate (GM), 2.5 mmol/L ADP, cytochrome c, 10 mmol/L succinate, 0.5 μmol/L rotenone, 2 μg/ml oligomycin, 2.5 μmol/L carbonyl cyanide p-trifluoromethoxyphenyl-hydrazone (FCCP) and 2.5 umol/L antimycin A.

### H_2_O_2_ Measurement

H_2_O_2_ production was measured in mitochondrial isolates from quadriceps by Amplex Red (Invitrogen corp., Carlsbad, CA, United States) using a spectrofluorometer (Horiba Scientific, Japan) as described previously (Zabielski et al., 2016). Briefly, generation of H_2_O_2_ was measured in mitochondrial isolates alone, and with the stepwise addition of 10 mmol/L glutamate/2 mmol/L malate (GM), 10 mmol/L succinate, 2.5 mmol/L ADP, 2 μg/ml oligomycin, 0.5 μmol/L rotenone, 2.5 μmol/L carbonyl cyanide p-trifluoromethoxyphenyl-hydrazone (FCCP) and 2.5 μmol/L antimycin A.

### Statistics

For all experiments other than transcriptomic and pathway analysis (described above), Graph Pad Prism software was used to determine significance. Student’s 2 tailed *t*-test was performed for the comparison of 2 groups. Two-way ANOVA was applied for the comparison of three or more groups with Tukey’s test for multiple comparisons.

## Results

### Muscle-Specific Deletion of Insulin Receptor or IGF-1 Receptor Alone Mildly Changes Gene Expression, Whereas Combined Loss of Insulin Receptor/IGF-1 Receptor Dramatically Alters the Muscle Transcriptome

**FIGURE 1:**
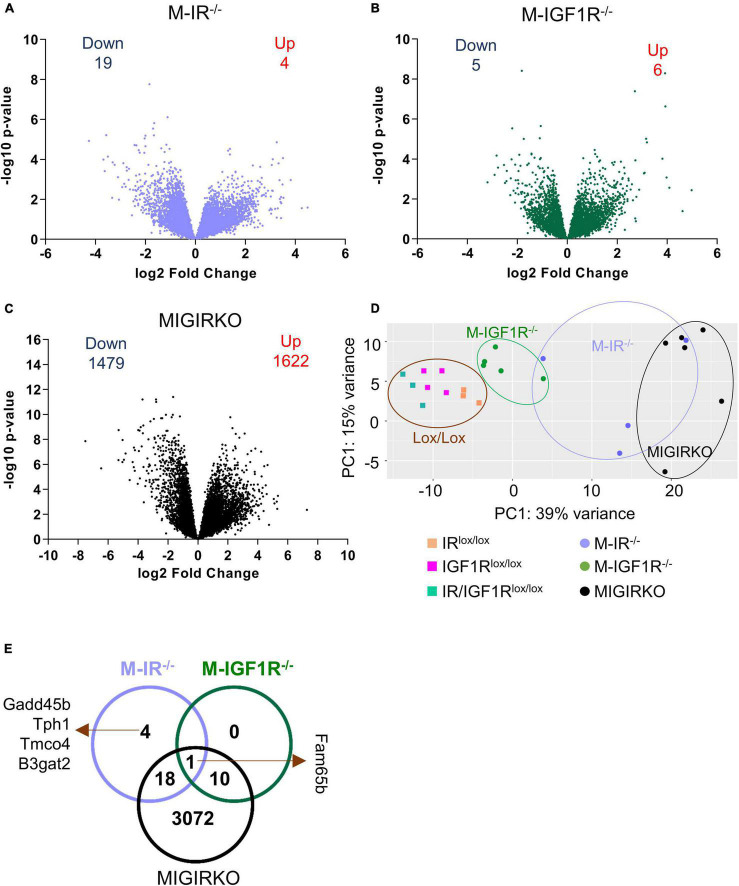
Muscle-specific deletion of insulin receptor (IR) or IGF-1 receptor (IGF1R) alone mildly changes gene expression, whereas combined loss of IR/IGF1R dramatically alters the muscle transcriptome. **(A–C)** Volcano plots depicting relative expression (log2 Fold Change) from RNA-Seq plotted against the −log10 *p*-Value for all identified genes in M-IR^–/–^ **(A)**, M-IGF1R^–/–^ **(B)**, and MIGIRKO **(C)** muscle compared with their respective lox/lox controls. **(D)** Principal component analysis (PCA) plot of RNA seq analysis from M-IR^–/–^, M-IGF1R^–/–^, and MIGIRKO muscle with their respective lox controls. **(E)** Venn diagram of differentially regulated genes among M-IR^–/–^, M-IGF1R^–/–^, and MIGIRKO muscle. (“Down” and “Up” numbers represent FDR < 0.05 and Fold Change ≥ |1.5| and excludes genes targeted for recombination such as Insr and Igf1r).

Muscle-specific loss of IGF1R (M-IGF1R^–/–^) has no effect, whereas loss of IR (M-IR^–/–^) causes a small decrease in muscle mass, and combined deletion of both IR and IGF1R (MIGIRKO) causes dramatic muscle atrophy (O’Neill et al., 2016). A similar pattern is seen with decreases in mitochondrial function in permeabilized soleus fibers using various substrates, including pyruvate, palmitoyl-carnitine, or glutamate in combination with malate (Bhardwaj et al., 2021). In the current study, we evaluated the relative contributions of IR and IGF1R in muscle to global gene expression using RNA-seq and the role of FoxO transcription factors in this process. Global transcriptomic profiling in mixed quadriceps and gastrocnemius muscles identified > 15,000 genes (Supplementary Table 1, Sheet 1), and these are shown as volcano plots for M-IR^–/–^, M-IGF1R^–/–^ and MIGIRKO compared with their floxed littermate controls (see methods for description of control mice) in Figures 1A–C, respectively. These show that loss of insulin receptor alone in muscle differentially altered (FDR < 0.05 and fold change ≥ |1.5|) the expression of only 23 genes, of which 4 (Ehd4, Irak2, Tmco4, and B3gat2) were upregulated and 19 genes were downregulated (Supplementary Table 1, Sheet 2). Likewise, deletion of IGF1R in muscle changed the expression of only 11 genes (Supplementary Table 1, Sheet 3), only 1 of which (the Rho family interacting protein Fam65b) overlapped with the muscle IR-regulated genes. By contrast, combined loss of IR and IGF1R in MIGIRKO, dramatically altered the expression of 3101 genes, among which 1622 were upregulated and 1479 genes were downregulated (Supplementary Table 1, Sheet 4), indicating the critical role provided by these two receptors to compensate for each other in control of gene expression. Principal component analysis (Figure 1D) of the combined RNA data showed that all lox/lox controls clustered together and were distinct from the MIGIRKO cluster, with the M-IGF1R^–/–^ and M-IR^–/–^ falling in between. Not surprisingly, comparison of differentially regulated genes among M-IR^–/–^, M-IGF1R^–/–^, and MIGIRKO (Venn diagram- Figure 1E) revealed that most of the genes altered by loss of IR or IGF1R alone were also regulated in MIGIRKO.

### Combined Loss of Insulin Receptor/IGF-1 Receptor in Muscle Downregulates Mitochondrial Pathways, Alters Fibrosis, Hypertrophic and Calcium/Other Signaling Pathways, and Impairs Mitochondrial Function

**FIGURE 2:**
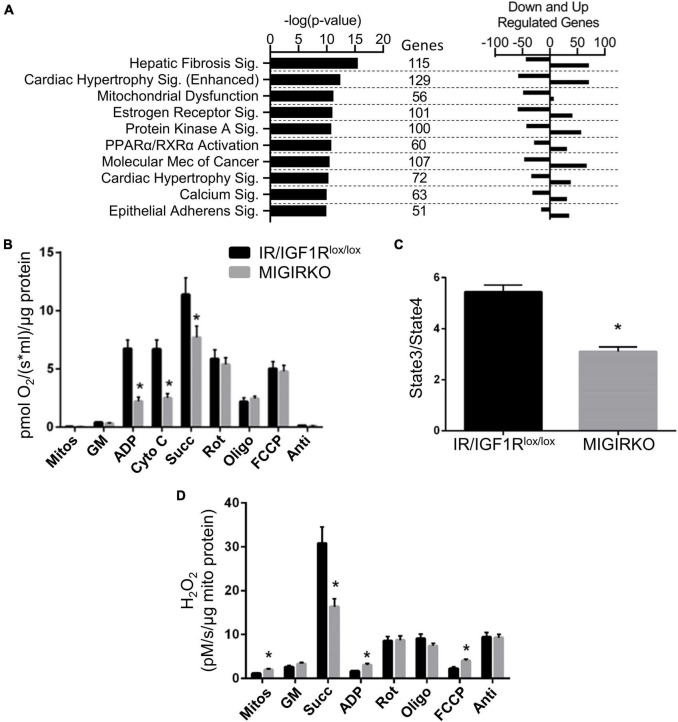
Combined loss of IR/IGF1R in muscle downregulates mitochondrial pathways, alters fibrosis, hypertrophic and calcium/other signaling pathways, and impairs mitochondrial function. **(A)** Canonical pathways in MIGIRKO muscle enriched from differentially regulated genes (FDR < 0.05 and Fold Change ≥ |1.5|) using Ingenuity Pathway Analysis. **(B)** Basal and maximal mitochondrial respiration in muscle mitochondria with glutamate/malate with subsequent addition of various substrates and inhibitors as indicated. **(C)** Respiratory control ratios (Glutamate/Malate/Succinate) measured in MIGIRKO and lox/lox controls. **(D)** H_2_O_2_ generation measured in mitochondrial isolates with sequential addition of substrates and mitochondrial inhibitors. (Mitos-Mitochondria, GM-Glutamate and Malate, cyto c-Cytochrome c, Succ-Succinate, Rot-Rotenone, Oligo-Oligomycin, FCCP-carbonyl cyanide p-trifluoromethoxyphenyl-hydrazone, and Anti-Antimycin A; Student *t*-test *p < 0.05, MIGIRKO vs. lox/lox controls).

The differentially regulated genes in MIGIRKO muscle were subjected to Ingenuity Pathway Analysis (Supplementary Table 2). The top 10 pathways and include fibrosis, hypertrophy, mitochondrial dysfunction, estrogen receptor-, PPAR/RXR-, protein kinase A-, and calcium-signaling (Figure 2A). While most of these pathways included genes that were both upregulated and downregulated in MIGIRKO muscle, among the 56 genes in the Mitochondrial Dysfunction category, 49 were downregulated in MIGIRKO muscle, consistent with the fact that downregulation of mitochondrial genes is a strong feature in muscle from patients with diabetes (Patti et al., 2003).

To determine the effect of this gene regulation on mitochondrial function, we measured mitochondrial respiration and H_2_O_2_ production in mitochondria isolated from MIGIRKO quadriceps muscle. This revealed that maximal respiration was significantly decreased with the addition of ADP in MIGIRKO muscle (Figure 2B), in agreement with our previous work (Bhardwaj et al., 2021). Addition of cytochrome c did not change respiration, confirming that the outer mitochondrial membrane was intact and not damaged by the isolation process. Subsequent sequential addition of succinate (complex-II substrate) and the mitochondrial inhibitors rotenone (complex-I), oligomycin (complex-V), FCCP (uncoupler), and antimycin A (complex-III) demonstrated that mitochondrial respiration using glutamate/malate substrates was markedly impaired in MIGIRKO compared with controls. Respiratory control ratio (State 3/State4) was decreased by ∼50% in MIGIRKO muscle when compared with muscle of the floxed controls (Figure 2C). H_2_O_2_ was increased in mitochondrial isolates of MIGIRKO muscle, and with the addition of ADP and FCCP, whereas H_2_O_2_ generation was decreased when the complex-II substrate succinate was added prior to ADP (Figure 2D), which may reflect a reduction in reverse electron transport due to decreased complex I function in MIGIRKO mitochondria (Robb et al., 2018; Bhardwaj et al., 2021).

### Muscle-Specific Deletion of FoxO1/3/4 Alters Amino Acid Degradation Genes, and Reverses Many of the Transcriptomic Changes After Combined Deletion of Insulin/IGF-1 Receptors

**FIGURE 3:**
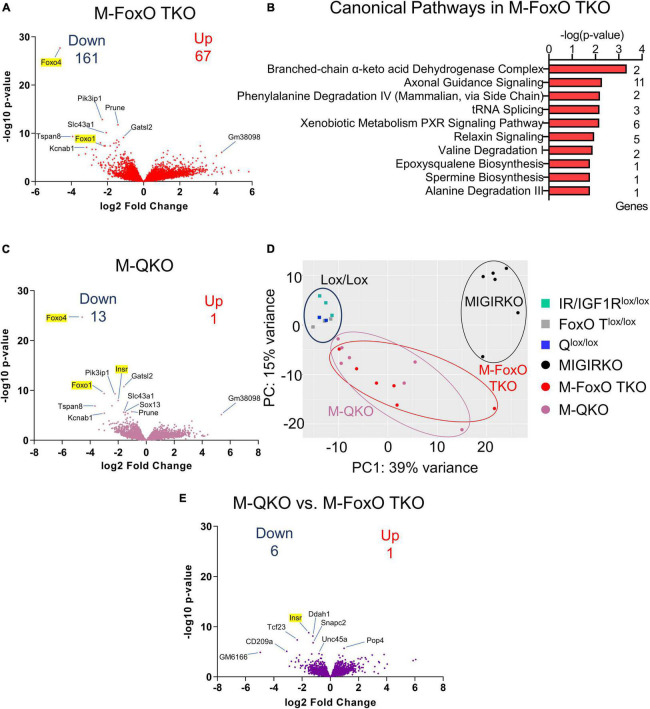
Muscle-specific deletion of FoxO1/3/4 alters amino acid degradation genes, and loss of FoxO1/3/4 along with IR/IGF1R in M-QKO reverses many of the transcriptomic changes in MIGIRKO. **(A)** Volcano plot of transcriptomic changes after triple knock out of FoxO1/3/4 in M-FoxO TKO muscle. **(B)** Canonical pathways enriched from differentially regulated genes in M-FoxO TKO muscle **(C)** Volcano plot of genes from M-QKO muscle. **(D)** Principal component analysis (PCA) plot of RNA seq data from MIGIRKO, M-FoxO TKO, and M-QKO muscle with their respective lox controls. **(E)** Volcano plot of transcriptomic changes between M-QKO and M-FoxO TKO muscle. (“Down” and “Up” represent FDR < 0.05 and Fold Change ≥ |1.5,| and excludes highlighted genes targeted for recombination: Insr, Igf1r, Foxo1, -3, -4).

FoxOs are critical mediators of insulin action on transcription in liver (Zhang et al., 2012; Sullivan et al., 2015) and have been shown to play a role in muscle atrophy associated with diabetes (O’Neill et al., 2019) and loss of IR and IGF1R (O’Neill et al., 2016). Deletion of FoxOs in muscle significantly altered the expression of 228 genes under normal fed conditions, among them 67 were upregulated, whereas 161 genes were downregulated (Supplementary Table 1, Sheet 5). Volcano plot of RNA seq data from M-FoxO TKO compared to FoxO1/3/4 triple floxed control (FoxO T^lox/lox^) mice is shown in Figure 3A. Ingenuity Pathway Analysis from the differentially regulated genes showed that the amino acid degradation and biosynthesis pathways were among the top hits in M-FoxO TKO muscle (Figure 3B). For example, gene branched chain ketoacid dehydrogenase E1, beta (Bckdhb) and dihydrolipoamide branched chain transacylase E2 (Dbt), two enzyme components involved in degradation of branched-chain amino acids and valine were decreased. Likewise, genes needed for phenylalanine and alanine degradation were decreased in M-FoxO TKO muscle, indicating that FoxOs are important for regulation of genes involved in amino acid metabolism under normal fed conditions. These pathways are distinct from FoxO-mediated regulation of protein degradation pathways identified under diabetic conditions (O’Neill et al., 2019).

To determine the contribution of FoxOs to the transcriptional changes in MIGIRKO, we performed RNA sequencing on M-QKO muscle. A volcano plot comparing M-QKO to Q^lox/lox^ controls reveals minimal differences in gene expression (Figure 3C). After eliminating genes that we targeted (Insr, Igf1r, Foxo1/3/4), only 14 genes were differentially regulated in M-QKO, among them 1 was upregulated, whereas 13 genes were downregulated (Supplementary Table 1, Sheet 6). The transcripts that were changed most significantly showed a high degree of overlap between M-QKO and M-FoxO TKO. We performed a PCA analysis that again showed that controls from the different colonies (IR/IGF1R^lox/lox^, FoxO T^lox/lox^, and Q^lox/lox^) cluster together, and these were very different from MIGIRKO, whereas M-FoxO TKO and M-QKO were distinct from these groups, but not from each other (Figure 3D). In an effort to determine if changes existed between M-QKO and M-FoxO TKO, we compared these two separate mouse lines to each other, although this has the significant limitation of mixed background strains (see methods). M-QKO vs. M-FoxO TKO showed only 7 genes were statistically different between the two knockout mouse lines, not including genes that we targeted (Insr, Igf1r, Foxo1/3/4) (Figure 3E and Supplementary Table 1, Sheet 7) and no differences were seen when Q^*lox*/*lox*^ and FoxO T^*lox*/*lox*^ were compared (Supplementary Table 1, Sheet 7). In summary, these results indicated that loss of FoxO1/3/4 along with IR/IGF1R in M-QKO reverses or normalized the expression of almost all genes that were altered in MIGIRKO muscle.

### FoxOs Regulate Genes in Oxidative Phosphorylation, Inflammatory Signaling, Protein Kinase A Signaling, and Cardiac Hypertrophy Pathways Downstream of Insulin/IGF-1 Receptors in Muscle

**FIGURE 4:**
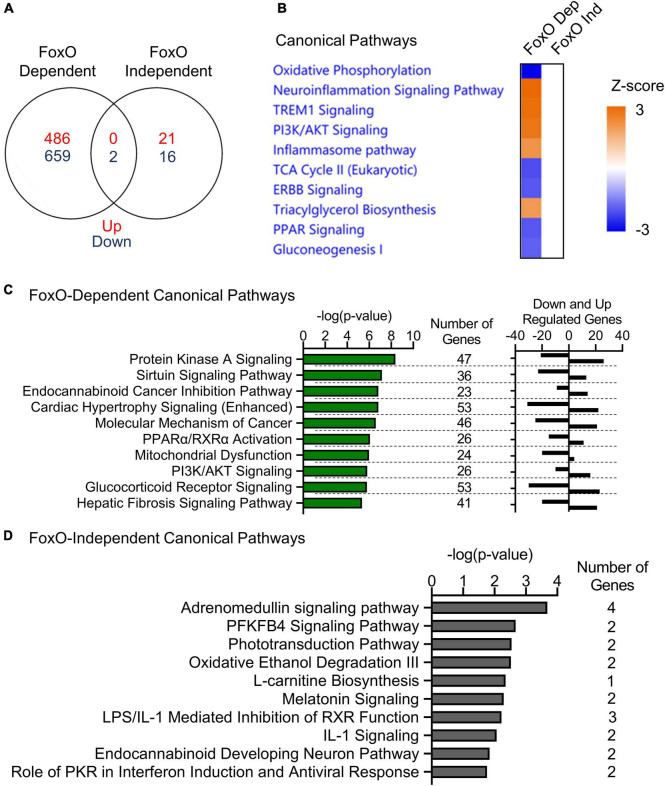
FoxOs proteins regulate genes in oxidative phosphorylation and inflammatory signaling pathways in MIGIRKO muscle. **(A)** Venn diagram of FoxO-dependent and FoxO-independent genes in muscle. **(B)** Canonical pathways enriched in comparison analysis of from FoxO-dependent and FoxO-independent genes by Ingenuity pathways analysis. **(C,D)** Canonical pathways enriched from FoxO-dependent **(C)** and FoxO-independent **(D)** genes by Ingenuity pathways analysis.

To determine which transcriptional changes in MIGIRKO were mediated by FoxO transcription factors, we compared fold changes of each gene between MIGIRKO vs. IR/IGF1R^lox/lox^ and M-QKO vs. M-FoxO TKO, and created a list of genes, whose expression is dependent and independent on FoxOs (Supplementary Table 3, Sheet 1, and section “Materials and Methods” for details). The comparison was designed this way to determine which genes/pathways changed in response to IR/IGF1R deletion in the presence or absence of FoxOs. “FoxO-dependent” were defined as genes where the log2 fold change (logFC) of MIGIRKO/IR/IGF1R^lox/lox^ was statistically greater than twice the magnitude of logFC M-QKO/M-FoxO TKO. This method was robust and revealed that genes such as Ehd4 and Ptpn3 were highly regulated in MIGIRKO, but not different between M-QKO and M-FoxO TKO (Supplementary Figures 1A,B). “FoxO-independent” genes were defined as those where the minimum t-statistic of MIGIRKO/IR/IGF1R^lox/lox^ and of M-QKO/M-FoxO TKO (if they were in the same direction) was greater than expected by chance. The analysis was done using the M-QKO/M-FoxO TKO in hopes of avoiding confounding by genes whose basal expression is regulated by FoxOs. Validation of this approach by selecting 2 of the top regulated genes, such as Map2k7 and Aldh9a1, showed that they were regulated in the same direction in MIGIRKO and in M-QKO compared to their controls, IR/IGF1R^lox/lox^ and M-FoxO TKO, respectively (Supplementary Figures 1C,D). The expression of 1147 genes in MIGIRKO muscle were dependent on FoxOs with an FDR of <0.05, among them 486 were upregulated, whereas 661 genes were downregulated (Figure 4A). Expression of only 39 genes in MIGIRKO were FoxO-Independent, in which 21 were upregulated, while 18 genes were downregulated.

When compared using Ingenuity Pathway Analysis, FoxO-dependent genes were most highly enriched for repression of the oxidative phosphorylation pathway (Figure 4B). Additionally, tricarboxylic acid cycle (TCA), ERBB and PPAR signaling, and gluconeogenesis were predicted as downregulated, whereas neuroinflammation signaling, TREM1 signaling, PI3K/AKT signaling, inflammasome pathway, and triacylglycerol biosynthesis were predicted as upregulated from FoxO dependent genes. When analyzed individually with IPA, the top FoxO dependent canonical pathways (Supplementary Table 3, Sheet 2) were most enriched for protein kinase A signaling, sirtuin signaling (which includes many OXPHOS and other mitochondrial genes), cancer-related, cardiac hypertrophy, PPARα/RXRα activation, and mitochondrial dysfunction (Figure 4C). By contrast, FoxO Independent genes revealed categories including adrenomedullin signaling, PFKFB4 signaling, phototransduction, oxidative ethanol degradation and L-carnitine biosynthesis pathways (Figure 4D).

**FIGURE 5:**
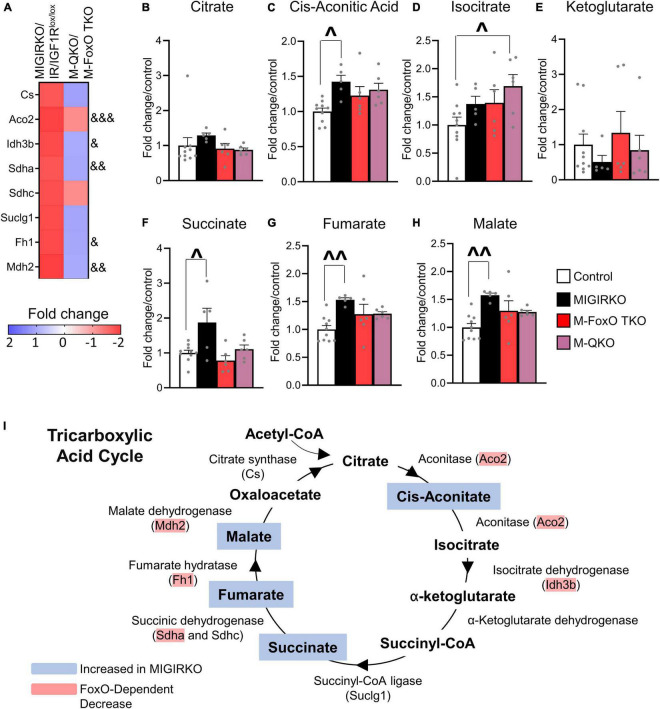
MIGIRKO muscle shows decreased TCA cycle genes and leads to TCA metabolite accumulation, which were normalized in M-QKO muscle. **(A)** Heat map of RNA-Seq results showing fold change of TCA cycle genes in MIGIRKO vs. IR/IGF1R^lox/lox^ and M-QKO vs. M-FoxO TKO. **(B–H)** Levels of TCA cycle metabolites measured in muscle from control, MIGIRKO, M-FoxO TKO, and M-QKO. **(I)** Schematic of tricarboxylic acid cycle showing regulation of genes and accumulation of metabolites in MIGIRKO. (& FDR < 0.05, && FDR < 0.01, &&& FDR < 0.001 MIGIRKO/IR/IGF1R^lox/lox^ vs. M-QKO/M-FoxO TKO; ^*p* < 0.05, ^^*p* < 0.01 as indicated with two-way ANOVA; number of animals used in metabolomic study *n* = 5–10).

We further investigated the repression of TCA cycle predicted by the FoxO dependent genes by extracting TCA cycle genes from RNA-Seq analyses and by measuring TCA metabolites. Eight TCA cycle genes (Cs, Aco2, Idh3b, Sdha, Sdhc, Suclg1, Fh1, and Mdh2) were significantly decreased in MIGIRKO and among these, expression of Aco2, Idh3b, Sdha, Fh1, and Mdh2 were dependent on FoxOs (Figure 5A). Quantification of TCA cycle metabolites was performed by mass spectrometry in MIGIRKO, M-FoxO TKO, and M-QKO and compared to controls (combined IR/IGF1R^lox/lox^, FoxO T^lox/lox^, and Q^lox/lox^). Citrate and alpha-ketoglutarate were unchanged across these groups (Figures 5B,E). We observed increases the level of cis-aconitic acid, succinate, fumarate, and malate in MIGIRKO muscle, whereas levels of these metabolites were unchanged in M-FoxO TKO and M-QKO muscle (Figures 5C,D,F–H). Schematically, the down regulation of Aco2, Sdha, Fh1, and Mdh2, agrees with the accumulation of their precursor metabolites (Figure 5I). Thus, in addition to regulation of OXPHOS genes (Bhardwaj et al., 2021), FoxOs also regulate TCA cycle genes and metabolites in MIGIRKO muscle.

### Deletion of Insulin/IGF-1 Receptors in Muscle Dysregulates Calcium Signaling Genes, and This Is Reversed by FoxO Deletion

**FIGURE 6:**
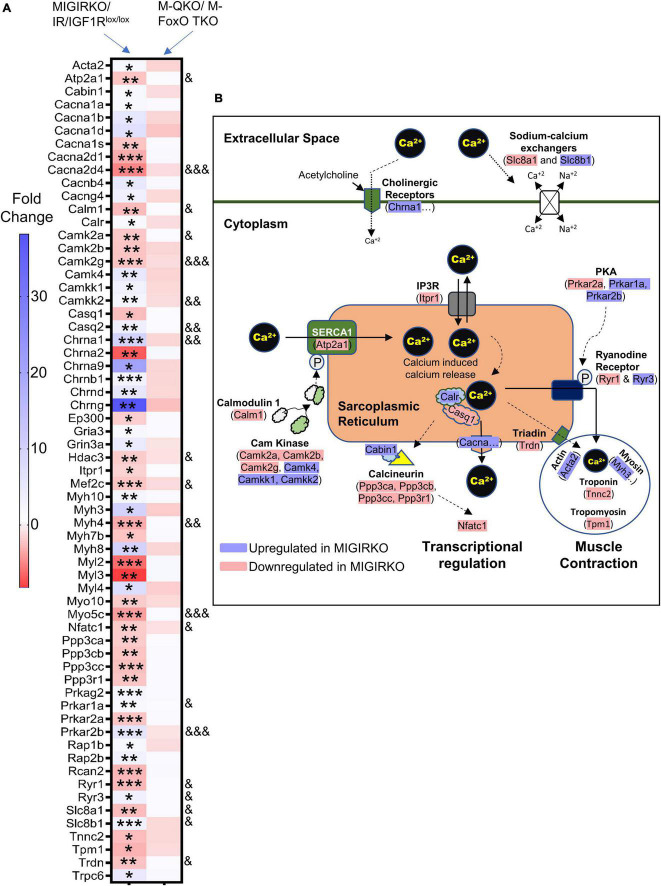
Calcium signaling was heavily perturbed in MIGIRKO from FoxO dependent genes, and the expression of these genes were normalized with the deletion of FoxOs in M-QKO muscle. **(A)** Heat map of calcium signaling genes in MIGIRKO vs. IR/IGF1R^lox/lox^ and M-QKO vs. M-FoxO TKO. **(B)** Schematic showing calcium signaling genes that are up-and down-regulated after loss of IR/IGF1R in muscle (Modified from ingenuity pathway analysis model). (*FDR < 0.05, **FDR < 0.01, ***FDR < 0.001 MIGIRKO vs. IR/IGF1Rl^ox/lox^;& FDR < 0.05, && FDR < 0.01, &&& FDR < 0.001 MIGIRKO/IR/IGF1R^lox/lox^ vs. M-QKO/M-FoxO TKO by “FoxO-dependent” analysis).

Ingenuity Pathway Analysis in MIGIRKO muscle showed that calcium signaling was highly enriched. Calcium homeostasis plays a critical role in mitochondrial function and muscle contractility (Guerrero-Hernandez and Verkhratsky, 2014; Vallejo-Illarramendi et al., 2014; Mosqueira et al., 2021), and may represent a unique pathway that has not been previously identified as a target of insulin/IGF-1 transcriptomic regulation. We extracted RNA-Seq data of 63 differentially regulated genes from MIGIRKO assigned to calcium signaling pathways and observed that many of these were classical calcium handing genes such as sarcoplasmic/endoplasmic reticulum calcium ATPase (SERCA), calcium/calmodulin dependent protein kinases (CamK), calcium sodium channels, inositol 1,4,5-triphosphate and ryanodine receptors (Figure 6A). Interestingly, some of the genes enriched in calcium signaling pathways were overlapped with the PKA and cardiac hypertrophic signaling, which were also identified in MIGIRKO pathway analyses. The expression of nearly one-third of the calcium signaling genes were statistically reversed in M-QKO using the “FoxO Dependent” analysis (Figure 6A). Among these FoxO dependent genes, seven were upregulated (Camkk2, Casq2, Chrna1, Prkar1a, Prkar2b, Ryr3, and Slc8a1), whereas 13 were downregulated (Atp2a1, Cacna2d4, Calm3, Camk2a, Camk2g, Hdac3, Mef2c, Myh4, Myo5c, Nfatc1, Ryr1, Slc8a1, and Trdn) in MIGIRKO muscle. Importantly, sarcoplasmic reticulum (SR) calcium ATPase 1 (Atp2a1), the major isoform of SERCA in skeletal muscle that controls transport of cytosolic Ca^2+^ into the SR, and ryanodine receptor 1 (Ryr1), the major Ryr in skeletal muscle that releases Ca^2+^ from SR, were decreased in MIGIRKO and reversed in M-QKO, indicating IR and IGF1R play a role in regulating sarcoplasmic calcium flux via FoxOs. A schematic of the major calcium signaling genes found in skeletal muscle reveals a predominant downregulation of calcium homeostasis when IR/IGF1R signaling is lost (Figure 6B).

## Discussion

Diabetes induces muscle weakness, contributes to physical disability, and increases the risk of morbidity and mortality. Insulin action on muscle increases glucose uptake, promotes protein anabolism, improves mitochondrial function, and regulates gene expression of various pathways, but how these are linked at a molecular level is not fully understood. In the current study, we investigated the contribution of FoxO transcription factors to the transcriptomic regulation of muscle genes by IR and IGF1R signaling using muscle specific knock-out mouse models. We showed that combined loss of IR/IGF1R in MIGIRKO altered > 3000 genes, related to multiple pathways including fibrosis, hypertrophy, and mitochondrial dysfunction. Furthermore, by combining IR/IGF1R knockout with knockout of the genes encoding all three FoxO proteins expressed in muscle, we show that nearly 97% of the IR/IGF1R regulated genes are downstream of FoxOs. Oxidative phosphorylation, TCA cycle, and inflammatory pathways were the top hits enriched from the FoxO-dependent genes. This is also reflected by the functional changes, with decreased mitochondrial respiration and increased H_2_O_2_ production in MIGIRKO muscle, which is reversed in M-QKO. Thus, activation of FoxO transcriptional activity in uncontrolled diabetes contributes to wide-ranging changes in muscle biology that ultimately results in diabetes-related muscle atrophy and weakness.

Numerous studies indicate that insulin action in muscle regulates a wide variety of physiological functions and controls transcripts of metabolism, autophagy, mitochondrial function, chromatin remodeling, and cytoskeletal maintenance (O’Brien and Granner, 1996; Rome et al., 2003; Batista et al., 2019; White and Kahn, 2021). The current study reveals that under normal physiological conditions (in the absence of systemic glucose or lipid abnormalities) loss of IR or IGF1R alone in muscle only mildly alter gene expression, consistent with the mild change in muscle mass from these animals (O’Neill et al., 2015). However, combined loss of IR/IGF1R dramatically changed the muscle transcriptome in MIGIRKO mice. Along with the broad downregulation of OXPHOS genes, this study also identified the repression of TCA cycle enzymes resulting in impaired mitochondrial respiration and accumulation of mitochondrial metabolites. These changes in the muscle transcriptome and metabolite levels fit well with our recently published results on mitochondrial function in permeabilized soleus fibers from M-IGF1R^–/–^, M-IR^–/–^, and MIGIRKO mice, which indicate the following: (1) deletion of IR alone in muscle mildly decreases mitochondrial function, whereas loss of IGF1R did not change mitochondrial respiration; (2) combined loss of both IR and IGF1R in MIGIRKO dramatically decreases mitochondrial respiration; and (3) these defects in MIGIRKO mitochondria were normalized in M-QKO muscle (Bhardwaj et al., 2021). Furthermore, this pattern in decline of mitochondrial function is also mimicked in muscle grip strength in these mice (O’Neill et al., 2016), indicating a strong correlation between mitochondrial function and strength regulation by insulin/IGF-1 action through FoxOs. PGC1α (Ppargc1a) and Nrf-1/2 are critical transcriptional regulators of mitochondria in muscle, but we did not find these pathways enriched in the current study, and our previous work showed no differences in PGC1α or Nrf expression in our various models (Bhardwaj et al., 2021; Supplementary Figure 3). Impairment of mitochondrial respiratory defects have been associated with insulin resistance, and both type 1 and type 2 diabetes, but the cause-and-effect relationship has been controversial. Studies in humans and mice have shown that insulin deficiency in type 1 and type 2 diabetes can drive mitochondrial abnormalities in muscle (Affourtit, 2016; Zabielski et al., 2016), while other studies indicated that diet induced obesity is not sufficient to cause mitochondrial decline (Franko et al., 2012; Toledo et al., 2018). Our study directly shows that a complete loss of insulin action by deletion of IR/IGF1R decreases mitochondrial respiration. Thus, the current study and our other recent work (O’Neill et al., 2016, 2019; Bhardwaj et al., 2021) revealed that FoxOs mediate the transcriptional downregulation of mitochondrial genes and that the decrease in mitochondrial function and can contribute to the decreased muscle strength and mass in diabetes.

At a mechanistic level, FoxO are mediators of diabetes-induced muscle atrophy, and other forms of acute muscle atrophy, by their effect to help regulate proteolysis (Sandri et al., 2004; Zhao et al., 2007; O’Neill et al., 2016, 2019). The current study also reveals that loss of FoxOs in muscle alters the expression of amino acid degradation pathways, particularly branch chain ketoacid dehydrogenase and valine degradation genes. Indeed, we previously observed that levels of valine, isoleucine, and leucine trended to be higher in muscle of M-FoxO TKO mice vs. controls, (O’Neill et al., 2019) but were even more markedly increased in streptozotocin-induced (STZ) diabetes, indicating other factors also influence amino acid levels in muscle. Additionally, our results were consistent with the finding that various amino acid catabolism genes are increased with overexpression of FoxO1 in the liver (Zhang et al., 2006). Thus, FoxOs are able to regulate genes involved in amino acid metabolism in muscle, but FoxOs alone do not control changes in muscle amino acid levels in response to STZ diabetes. Further studies are required to determine the coordination of FoxO-regulated muscle atrophy pathways and amino acid biosynthesis and degradative pathways.

Studies both in human and animals have shown that calcium signaling is impaired in insulin resistant and diabetic muscle, and calcium homeostasis is important for the muscle contraction, relaxation, and proper mitochondrial functioning (Lanner et al., 2006; Guerrero-Hernandez and Verkhratsky, 2014; Vallejo-Illarramendi et al., 2014; Agrawal et al., 2018; Mosqueira et al., 2021). FoxO3 controls mitochondrial calcium homeostasis in phenylephrine-stressed adult cardiomyocytes and in a rat model of heart failure with preserved ejection fraction (Chaanine et al., 2016), but the direct role of FoxOs on skeletal muscle calcium signaling is not well studied. We show that the major skeletal muscle isoforms of the SERCA pump (Atp2a) and ryanodine receptor (Ryr1) are downregulated in MIGIRKO in a FoxO-dependent manner, which may contribute to the severe muscle weakness in these animals. Calcium signaling via CAM kinase II and PKA signaling were also disrupted in MIGIRKO muscle with downregulation of some isoforms (Camk2a, -2b, -2g, and Prkar2a) and upregulation of others (Camkk, Camk4, Prkar1a and Prkar2b). Ppp3ca (also known as calcineurin A alpha), which plays an essential role in the transduction of intracellular calcium mediated signals, is downregulated in MIGIRKO and normalized with the deletion of FoxOs in M-QKO mice. The interaction between calcineurin action and FoxOs may thus form a feedback loop, given that overexpression of a calcineurin isoform CnAbeta1 in C2C12 myotubes inhibits FoxO and prevent muscle atrophy (Lara-Pezzi et al., 2007). Thus, FoxOs may also regulate muscle strength by controlling calcium signaling genes, and open avenues to study the mechanisms how IR/IGF1R and FoxO axis regulate calcium homeostasis in diabetes-related muscle atrophy.

There are several limitations to our study, the most prominent being the use of a mixed background strain, rather than back-crossing onto a pure genetic background. It is well established that background strain can cause significant variability in metabolic and transcriptomic studies (Fontaine and Davis, 2016; Lilue et al., 2018). Practically speaking, we were unable to backcross the QKO line since it has 11 alleles (Acta-Cre and 10 targeted floxed alleles in 5 genes), but these mice were generated at the same time as the M-FoxO TKO and do demonstrate very similar transcriptional regulation. We are also reassured that the primary findings that transcriptomic changes in mitochondrial pathways of MIGIRKO muscle was matched by mitochondrial dysfunction and metabolite accumulation, both in this project and our previous work (Bhardwaj et al., 2021), but it does not change that fact that genetic heterogeneity may contribute to variability in our findings and is a significant limitation. Additionally, muscle mitochondrial function is tightly connected to physical activity. Although we did not assess activity or submit these mice to exercise prior to mitochondrial studies, we have reported that spontaneous activity and free wheel running of MIGIRKO was decreased compared to controls (O’Neill et al., 2015, 2016). The mitochondrial defects in MIGIRKO may be partly due to inactivity, but were completely restored after knockout of FoxOs.

## Conclusion

**FIGURE 7:**
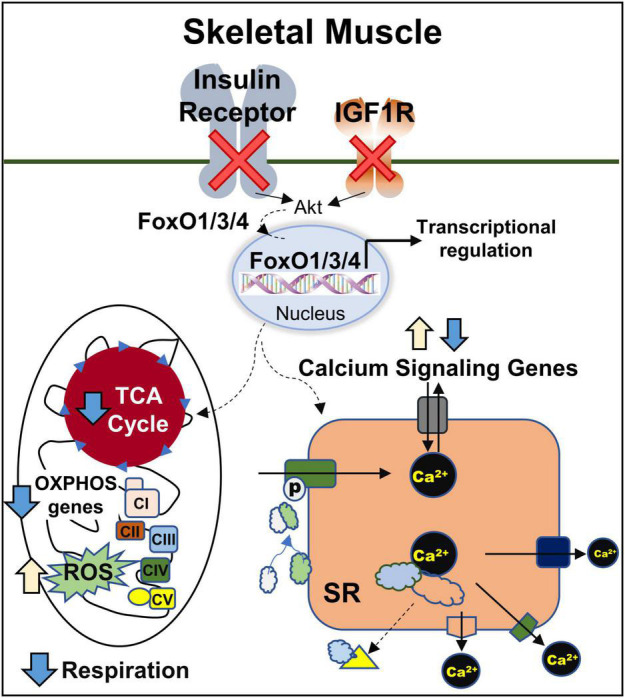
Regulation of mitochondrial and calcium signaling genes in muscle by IR/IGF1R is FoxO-Dependent. CI, CII, CIII, CIV, and CV, complexes I, II, III, IV, and V; OXPHOS, oxidative phosphorylation; ROS, reactive oxygen species; SR, sarcoplasmic reticulum; TCA, tricarboxylic acid.

In conclusion, our results identify the important role of FoxOs in insulin/IGF-1 signaling to regulate the gene expression in muscle and the impact of these changes on various metabolic pathways particularly TCA cycle, oxidative phosphorylation, and calcium signaling (Figure 7). Future studies will be helpful in defining the precise mechanisms by which FoxOs regulate mitochondrial respiration, ROS generation and calcium signaling.

## Data Availability Statement

RNA sequencing results have been published previously (O’Neill et al., 2019; Bhardwaj et al., 2021) and are publicly available at Gene Expression Omnibus database (GSE178356 and GSE136948).

## Ethics Statement

The animal study was reviewed and approved by the Institutional Animal care and Use Committee (IACUC) at both the University of Iowa and Joslin Diabetes Center.

## Author Contributions

GB designed the study, researched the data, and wrote the manuscript. CP, KK, and EW researched the data and helped to design experiments, analyzed the data, and wrote the manuscript. HP and JD performed the analysis on RNA-Seq data and included the design of the FoxO-dependent and independent studies. KN and CK provided reagents and helped to design experiments and to wrote the manuscript. BTO designed the study and helped to write the manuscript. All authors contributed to the article and approved the submitted version.

## Conflict of Interest

The authors declare that the research was conducted in the absence of any commercial or financial relationships that could be construed as a potential conflict of interest.

## Publisher’s Note

All claims expressed in this article are solely those of the authors and do not necessarily represent those of their affiliated organizations, or those of the publisher, the editors and the reviewers. Any product that may be evaluated in this article, or claim that may be made by its manufacturer, is not guaranteed or endorsed by the publisher.
