# Supplementary figures and images for: Transcriptomic Regulation of Muscle Mitochondria and Calcium Signaling by Insulin/IGF-1 Receptors Depends on FoxO Transcription Factors
Source: Front Physiol. 2022 Feb 4;12:779121. doi: 10.3389/fphys.2021.779121 (PMC8855073; doi:10.3389/fphys.2021.779121)

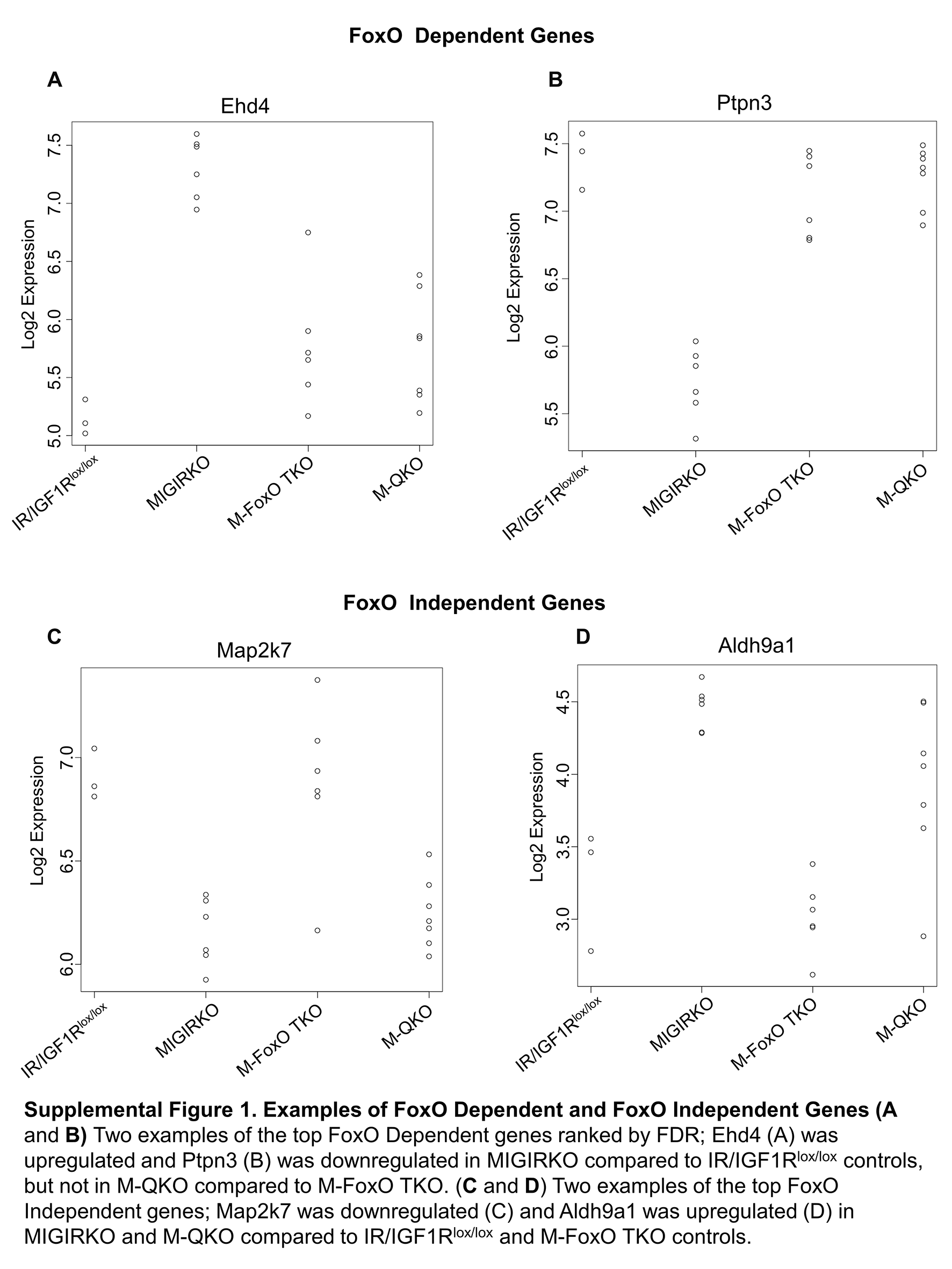

Supplement: Supplementary file 1 [file Image_1.tif]
